# Comparison of usual podiatric care and early physical therapy intervention for plantar heel pain: study protocol for a parallel-group randomized clinical trial

**DOI:** 10.1186/1745-6215-14-414

**Published:** 2013-12-03

**Authors:** Shane M McClinton, Timothy W Flynn, Bryan C Heiderscheit, Thomas G McPoil, Daniel Pinto, Pamela A Duffy, John D Bennett

**Affiliations:** 1 Rocky Mountain University of Health Professions, 561 East 1860 South, Provo, Utah 84606, USA; 2 Department of Physical Therapy, Des Moines University, 3200 Grand Avenue, Des Moines, IA 50312, USA; 3 Department of Physical Therapy, Rocky Mountain University of Health Professions, 561 East 1860 South, Provo, Utah 84606, USA; 4 Department of Orthopedics and Rehabilitation, University of Wisconsin – Madison, 1300 University Avenue, Madison, Wisconsin 53706, USA; 5 School of Physical Therapy, Rueckert-Hartman College of Health Professions, Regis University, 3333 Regis Boulevard, Denver, Colorado 80221, USA; 6 Department of Physical Therapy and Human Movement Sciences/Center for Healthcare Studies, Feinberg School of Medicine, Northwestern University, 645 North Michigan Avenue, Suite 1100, Chicago, Illinois 60611, USA; 7 Public Health Program, Des Moines University, 3200 Grand Avenue, Des Moines, Iowa 50312, USA; 8 Podiatric Medicine Program, Des Moines University, 3200 Grand Avenue, Des Moines, Iowa 50312, USA

**Keywords:** Cost, Exercise therapy, Musculoskeletal manipulations, Plantar fasciitis, Podiatry

## Abstract

**Background:**

A significant number of individuals suffer from plantar heel pain (PHP) and many go on to have chronic symptoms and continued disability. Persistence of symptoms adds to the economic burden of PHP and cost-effective solutions are needed. Currently, there is a wide variation in treatment, cost, and outcomes of care for PHP with limited information on the cost-effectiveness and comparisons of common treatment approaches. Two practice guidelines and recent evidence of effective physical therapy intervention are available to direct treatment but the timing and influence of physical therapy intervention in the multidisciplinary management of PHP is unclear. The purpose of this investigation is to compare the outcomes and costs associated with early physical therapy intervention (ePT) following initial presentation to podiatry versus usual podiatric care (uPOD) in individuals with PHP.

**Methods:**

A parallel-group, block-randomized clinical trial will compare ePT and uPOD. Both groups will be seen initially by a podiatrist before allocation to a group that will receive physical therapy intervention consisting primarily of manual therapy, exercise, and modalities, or podiatric care consisting primarily of a stretching handout, medication, injections, and orthotics. Treatment in each group will be directed by practice guidelines and a procedural manual, yet the specific intervention for each participant will be selected by the treating provider. Between-group differences in the Foot and Ankle Ability Measure 6 months following the initial visit will be the primary outcome collected by an independent investigator. In addition, differences in the European Quality of Life – Five Dimensions, Numeric Pain Rating Scale, Global Rating of Change (GROC), health-related costs, and cost-effectiveness at 6 weeks, 6 months, and 1 year will be compared between groups. The association between successful outcomes based on GROC score and participant expectations of recovery generally, and specific to physical therapy and podiatry treatment, will also be analyzed.

**Discussion:**

This study will be the first pragmatic trial to investigate the clinical outcomes and cost-effectiveness of ePT and uPOD in individuals with PHP. The results will serve to inform clinical practice decisions and management guidelines of multiple disciplines.

**Trial registration:**

ClinicalTrials.gov: NCT01865734

## Background

Plantar heel pain (PHP), or plantar fasciitis, is a common condition that approximately 1–2 million Americans receive treatment for each year [1,2]. Individuals with PHP commonly have prolonged and recurrent symptoms that cost from $192 to $396 million to treat annually [3-7]. Actual healthcare costs for PHP management are likely higher because this estimate reflects only 818,000 of the 1–2 million individuals estimated to have PHP annually and does not include indirect costs, such as loss of work productivity or costs associated with treatment provided by podiatrists and physical therapists [2].

The etiology of PHP is multifactorial including inflammation, chronic tendinopathy, foot posture or mobility dysfunction, lower leg inflexibility, mobility impairments of the foot, ankle, knee and hip, and neurodynamic dysfunction [8-17]. Due to the multifactorial nature of PHP, many terms such as plantar fasciitis, plantar fasciopathy, and plantar fasciosis have been used to describe the condition. Furthermore, there are a number of different treatments proposed to be effective in PHP and multiple healthcare providers that offer primary care for PHP [18-20].

Providers within the healthcare system that manage PHP include podiatrists, physical therapists, chiropractors, primary care physicians, and orthopedic surgeons. Prevalence estimates for individuals with PHP that seek care from non-physician providers who manage PHP is not available. However, approximately 1 million visits for PHP were made each year to office or hospital-based physicians between the years 1995 and 2000 [2]; 62% of visits were made to primary care physicians and 31% to orthopedic surgeons, with physical therapy ordered or provided at 19% of these visits [2]. In addition to physicians, there is evidence to suggest that individuals with PHP also visit podiatrists and physical therapists frequently as primary providers for this problem. It has been estimated that up to 15% of patients seeking care from a podiatrist have a chief complaint of PHP and a survey of podiatrists indicated that PHP was the most prevalent condition in podiatry practice [21,22]. A survey of physical therapists in orthopedic practice indicated that PHP was the most common foot condition seen [23]. While physicians, podiatrists, and physical therapists each see a significant number of individuals with PHP, the primary role of several different disciplines may contribute to the observed variance in PHP management [2].

Clinical guidelines can provide direction in the presence of practice variation and multiple treatment options. Currently there are two clinical guidelines on PHP provided by the American College of Foot and Ankle Surgeons (ACFAS) and the Orthopaedic Section of the American Physical Therapy Association (APTA) [19,20]. Both guidelines are intended for use by podiatry and physician specialties, although the APTA guidelines are also intended for use by physical therapists [24]. While there are many common recommendations between the two guidelines, including foot and lower leg stretching, taping, orthotics, and night splints, there is an apparent difference in recommendations for the timing of referral to a physical therapist and inclusion of common physical therapy interventions for PHP. ACFAS guidelines recommend 6 weeks of unresponsive treatment and testing to include imaging (e.g., radiographs), corticosteroid injections, orthotics, taping, oral anti-inflammatories, and an unsupervised home program before referral to a physical therapist. In contrast, APTA guidelines do not indicate a timeline for certain interventions as described in the ACFAS guidelines. Additionally, recommendations for iontophoresis, manual therapy, and supervised exercise are included in the APTA guidelines without specific mention in the ACFAS guidelines. Since the APTA guidelines were written in 2008, additional evidence has been published supporting the use of manual therapy interventions in the management of PHP [10,25,26]. The conflicting recommendations of the ACFAS and APTA guidelines may result in different healthcare utilization patterns. Following the ACFAS recommendations, there is likely to be greater utilization of corticosteroid injections, oral medication, and radiographs in the first 6 weeks whereas a greater utilization of manual therapy and exercise procedures may result from following the APTA guidelines and recent manual therapy evidence. The discrepancies between guidelines and discipline-specific practice patterns for PHP contribute to uncertainty in the best management strategy of primary providers for PHP. An important factor to clarify is the role of physical therapy intervention within the first 6 weeks that a patient presents to a provider for PHP.

Early physical therapy intervention versus a delayed referral has demonstrated improved outcomes, decreased likelihood of surgery and injections, fewer office visits, and reduced advanced imaging and medication in individuals with low back pain [27-29]. No investigations were found on the impact of early physical therapy intervention in PHP. If physical therapy intervention provided early after initial presentation to a provider for PHP can reduce utilization of healthcare while improving outcomes, this would be a significant contribution to the economic and disability-related burden of PHP. Therefore, the purpose of this investigation is to compare the outcomes and costs associated with usual podiatric care (uPOD) versus early physical therapy intervention (ePT) in patients with PHP.

### Specific aims

Aim 1: Compare the difference in outcome between ePT versus uPOD. The null hypothesis that no difference in the Foot and Ankle Ability Measure (FAAM) between ePT and uPOD in individuals with PHP at the 6-month follow-up will be tested using a randomized clinical trial.

Aim 2: Compare the difference in secondary outcome measures using the 3-item (average, worst, least) numeric pain rating scale (NPRS), and global rating of change (GROC) scale. These measures will be taken concurrently with the FAAM in the randomized clinical trial to test the null hypothesis that there is no difference in NPRS and GROC between ePT and uPOD.

Aim 3: Compare the cost-effectiveness of ePT versus uPOD. Costs and quality of life measures will be captured concurrently in the randomized clinical trial to calculate incremental cost-effectiveness ratios and test the null hypothesis that there is no difference in cost-effectiveness between ePT and uPOD.

Aims 1 and 2 will be the focus of this paper although Aim 3 will also be considered in the planning of this project and is included here for clarity of the entire project’s scope. The procedures to accomplish Aim 3 will be outlined in a subsequent publication.

## Methods

### Study design

**Figure 1:**
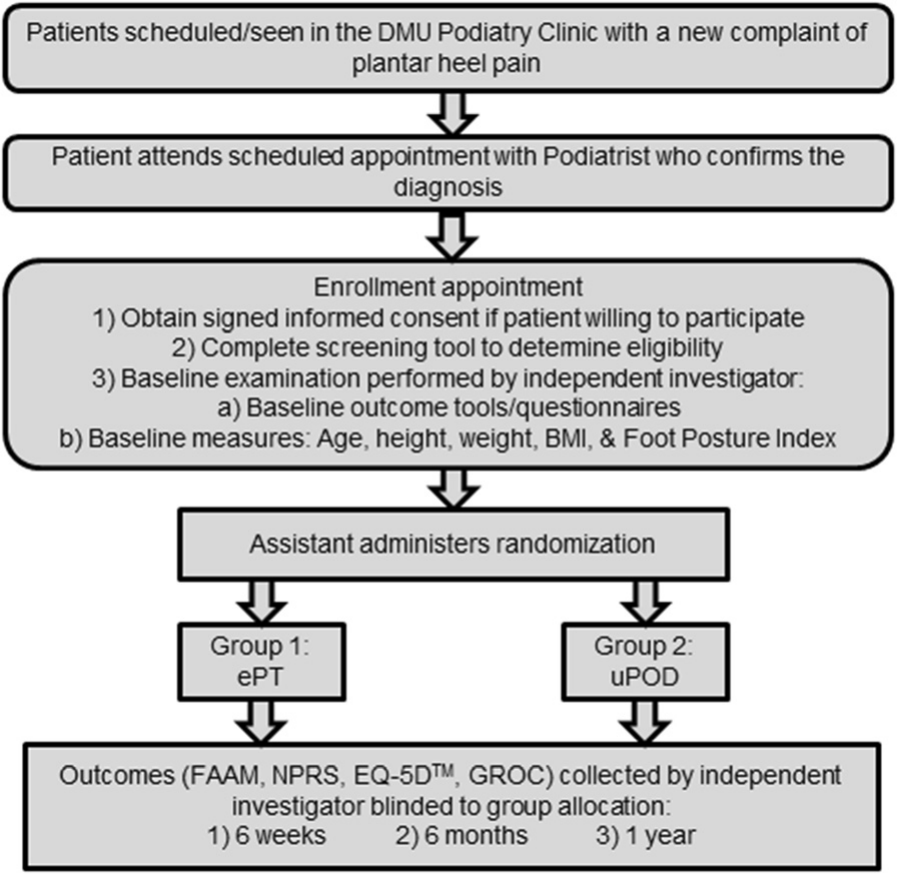
**Diagram of participant flow through the study.** BMI: Body mass index; DMU: Des Moines University; ePT: Early physical therapy intervention; EQ-5D^TM^: European Quality of Life – Five Dimensions; FAAM: Foot and Ankle Ability Measure; GROC: Global rating of change; NPRS: Numeric pain rating scale; PI: Principle investigator; uPOD: Usual podiatric care.

This investigation will be a block randomized clinical trial comparing participant-reported outcomes and cost-effectiveness of ePT and uPOD with follow-up extending to 1 year (Figure 1).

### Setting and ethical approval

The location for this study will be the Podiatry and Physical Therapy Departments at the Des Moines University Clinic, Des Moines, IA, USA. This study has received Institutional Review Board (IRB) Approval from Des Moines University (IRB identification, 04-13-02) and Rocky Mountain University of Health Professions (IRB identification 131034–02). Within the Des Moines University IRB approval, agreements regarding review of protected health information in preparation for research, and extraction of data from electronic health records were made between the Des Moines University Clinic and the principle investigator. As a part of this agreement, all enrolled participants will sign a Health Insurance Portability and Accountability Act of 1996 authorization to use and disclose individual health information for research purposes form in addition to the informed consent form.

### Participants

Individuals presenting with a primary complaint of PHP to the Podiatry Department of the Des Moines University Clinic will be recruited for this study. Participants will be included if they have a primary clinical diagnosis of PHP determined by having tenderness to palpation of the plantar aspect of the heel and pain associated with first step after waking in the morning or with progression of weight-bearing during the day. Eligible participants will be between the ages of 18–60 and demonstrate a score of less than 74/84 on the FAAM activities of daily living (ADL) subscale to allow for improvement of at least the minimum clinically important difference [30]. Participants will be excluded if they had prior surgery of the foot, ankle, or lower leg; clinical signs of radiculopathy; contraindications to manual therapy interventions (i.e., tumor, fracture, rheumatic inflammatory disease [rheumatoid, reactive and psoriatic arthritis, inflammatory bowel disease, ankylosing spondylitis, and systemic lupus erythematosus], osteoporosis, prolonged history of steroid use, severe vascular disease, etc.); clinical indication of plantar fascia rupture; are unable to complete questionnaires; unable or unwilling to comply with treatment recommendations of either treatment group; or had prior treatment for PHP in the past 6 weeks. In addition, individuals who have a body mass index (BMI) greater than 30 kg/m^2^ or symptoms for longer than 1 year will be excluded due to the greater risk of chronic symptoms and poor response to conservative management [4,6,13,31].

The number of participants for this study is based on the power needed to detect between-group differences in the primary outcome measure, the FAAM, at 6 months. In the absence of research comparing physical therapy and podiatry interventions or podiatry interventions using the FAAM as an outcome measure, sample size calculations are based on achieving a clinically meaningful difference between the groups and details from a recent clinical trial by Cleland et al. [10] that used similar methods to this investigation. Sample size estimate was made using G*Power 3.1.5 based on detecting a difference between groups greater than the minimal clinically important difference (MCID) (i.e., 9 point change) of the FAAM at 6 months with an alpha level of 0.05, 80% power, and pooled sample variance of 14.5 from Cleland et al. [10,30,32]. This resulted in an effect size of 0.62 and 42 participants needed per group. To account for participants who drop-in or drop-out of treatment, in addition to the possibility of some participants not returning the FAAM questionnaire, the sample will be increased by 33% resulting in 56 participants per group. The focus, and consequently the power analysis of this investigation is on functional outcome (based on FAAM scores) and therefore may result in underpowered analysis of secondary variables.

### Outcome measures

Outcomes will be collected at 6 weeks, 6 months, and 1 year after initial presentation. An investigator blinded to group allocation will collect all primary and secondary outcome measures. A small financial incentive will be provided to facilitate completion of outcome measures over the study duration.

#### Primary outcome measure

The FAAM ADL subscale will be used to assess participant-reported functional outcome. The FAAM has demonstrated an intraclass correlation coefficient of 0.89 and a MCID of 8 for the ADL subscale [30].

#### Secondary outcome measures

A 3-item, 0–10 (0 = no pain, 10 = worst imaginable pain), NPRS will be used to assess average pain in addition the lowest and highest levels of pain in the past week. The average of the three ratings will be used to represent overall pain intensity. The 3-item NPRS has demonstrated test-retest reliability of 0.61, and an MCID of 2 points [33]. The GROC scale will be used to assess participant-reported improvement. The GROC is a single question item that includes 15 ranks for global improvement from −7 (a very great deal worse) to 0 (about the same) to +7 (a very great deal better) [34]. Scores of +5 have previously been used as an indicator of clinical success [10,35]. Overall health status will be measured by the European Quality of Life-Five Dimensions (EQ-5D™)*.* The EQ-5D™ is a general measure of health-related quality of life that includes five items related to mobility, self-care, usual activities, pain/discomfort, and anxiety/depression [36]. Index scores from the United States general population will be applied to the EQ-5D™ to calculate the quality-adjusted life year, where 0.0 = death and 1.0 = perfect health [37].

### Intervention

All patients will attend one visit with a podiatrist where the podiatrist will perform an evaluation and provide intervention based upon usual practice patterns. After this visit, the patient will be invited to participate in the study and undergo a baseline examination that will collect data on variables indicated by Table 1. A member of the office staff will randomize group assignment using random block sizes of 4 and 6, and concealed envelopes containing group assignment to ePT or uPOD (Figure 1).

**Table 1:** Baseline measures of participant characteristics

**Characteristic**	**ePT**	**uPOD**
Age (years)		
Sex		
Height (cm)		
Weight (kg)		
BMI (kg/m^2^)		
Bilateral symptoms (%)		
Prior history of PHP (yes/no)		
Duration of symptoms (days)		
Foot Posture Index [38]		
Number of hours on feet/week		
NPRS		
FAAM		
EQ-5D™		
Healthcare resource use (US dollars)*		
General recovery expectation^†^		
Expectation for physical therapy^‡^		
Expectation for podiatry^‡^		
Treatment Preference (%):		
Neutral		
Physical Therapy		
Podiatry		

*Data derived from a participant-reported questionnaire. ^†^Measured by rating expected recovery according to the GROC scale [34]. ^‡^As measured on a 0–10 visual analog scale where 0 = not helpful and 10 = extremely helpful relative to plantar heel pain. BMI: Body mass index; ePT: Early physical therapy intervention; EQ-5D™: European Quality of Life-Five Dimensions; FAAM: Foot and Ankle Ability Measure; NPRS: Numeric pain rating scale; PHP: Plantar heel pain; uPOD: Usual podiatric care.

#### Early physical therapy intervention group (ePT)

Individuals in the ePT will be managed by a physical therapist in accordance with the APTA plantar heel pain practice guidelines and recent evidence in support of manual therapy intervention [10,19,25,26]. Treatment provided will be based on identified impairments and may include manual therapy (joint and soft tissue mobilization/thrust manipulation to the lower half of the body), lower leg and plantar foot specific stretching/self-mobilization, foot and lower leg muscle performance training, night splints, taping, over-the-counter orthotics/heel cup/heel cushion, and iontophoresis. The specific intervention will be selected at the discretion of the treating physical therapist, yet will be guided by a manual of procedures that directs intervention priorities and progression based on current best evidence (Tables 2 and 3). The principle investigator will conduct training sessions with each ePT provider to assure comprehension of the items and interventions included in the manual of procedures. Training sessions are estimated to last 1–4 hours per provider and will be conducted in brief sessions with follow-up until each therapist reports comprehension. Participants in the ePT group will be managed by the physical therapist without additional podiatric intervention until the participant achieves a GROC of “quite a bit better” and demonstrates independence in condition management strategies or a plateau in progress demonstrated by GROC or FAAM scores. While participants in the ePT group will be managed primarily by the physical therapist, they are free to return to the podiatrist if they choose.

**Table 2:** Physical therapist guide for intervention delivered to the early physical therapy intervention group

**Intervention category**	**Initial tier interventions**	**Second tier interventions**
Exercise	Stretching/mobility: Plantar fascia-specific, ankle dorsiflexion (knee bent and straight), self-lateral heel glide [10,19]	Stretching/mobility of the posterior thigh [39-41], and identified impairments of areas proximal to the lower leg
		Muscle performance training of foot and lower leg [12,42-47], in addition to identified impairments of proximal regions [48,49]
Manual therapy	Impairment based treatment directed at the ankle and foot [10,25,26]	Impairment based treatment directed at regions proximal to the ankle and foot including neurodynamic impairments [10,50,51]
Modalities	Iontophoresis with dexamethasone in cases with highly irritable and acute symptoms [19]	–
Tape/Orthotics	If participants do not present with an orthotic and did not receive one from their podiatry visit, the Treatment Direction Test [52] will be used for short-term pain relief and orthotic consideration.	–
Night splint	If symptoms persist for >6 months and this has not been tried previously [19]	–
Education	Preliminary information about plantar heel pain including prognosis, home program, and modifiable contributing factors (e.g., footwear, body weight, flexibility, foot loading/weight-bearing)	Brief pain neuroscience if central sensitization or peripheral neuropathic pain mechanisms are identified [53,54]
Home program	Home program will include less than 5 of Tier 1 or 2 exercises to facilitate adherence [55]	

**Table 3:** Phases and progression of physical therapy treatment

**Phase**	**Goals**	**Criteria for advancement to next phase**	**Interventions**
Phase I	1. Decrease irritability	1. Mild to moderate pain	1. Address contributing factors (footwear, orthotics, taping, neurodynamic impairments)
	2. Educate participant on condition and rehabilitation	2. Dorsiflexion ≥10 degrees (measured in prone with knee extended) [11], or symmetrical dorsiflexion to uninvolved side	
			2. Participant education
	3. Improve dorsiflexion		3. Modalities
			4. Stretching/self-mobilization
			a. Home Program; <5 exercises [55]
			b. Night splint (if symptoms persist for >6 months) [19]
			5. Manual therapy
Phase II	1. Further reduction in pain	1. Minimal to no pain	1. Exercise*
		2. Single leg heel raise ≥12 repetitions [56], or symmetrical performance to the uninvolved side	2. Manual therapy*
	2. Restore muscle performance		3. Gait training
	3. Minimize gait deviations		
		3. Walking items on FAAM ≤ “slight difficulty”	
	4. Enhance basic function(s)		
Phase III	1. Enhance higher level function(s) including sport and recreational activities	*Discharge when:*	1. Progression of exercise
		GROC ≥ “quite a bit better” and participant demonstrates understanding of independent condition management	2. Sport/recreation specific training
			2. Education on independent condition management and prevention
	2. Prevent recurrence		
		OR	
				Plateau evident in GROC or FAAM scores and participant demonstrates understanding of independent condition management

*Manual therapy during Phase II will address residual impairments from Phase I but Phase II treatment will reflect greater volume of exercise interventions than manual therapy compared to Phase I. FAAM: Foot and Ankle Ability Measure; GROC: Global rating of change scale.

#### Usual podiatric care group (uPOD)

**Figure 2:**
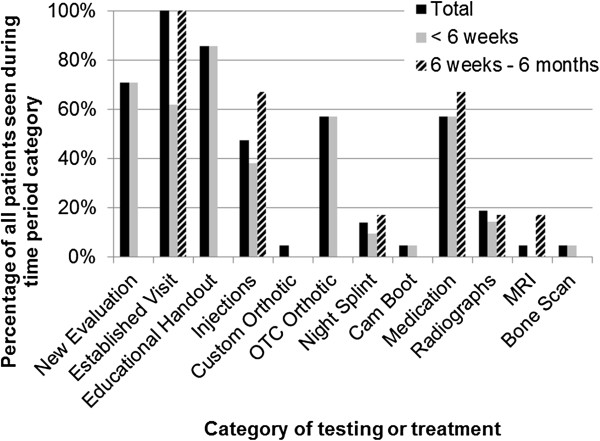
**Usual podiatric care (uPOD) represented by the percentage of patients and time relative to initial presentation of podiatric intervention or testing by podiatrists at the study location.** MRI: Magnetic resonance imaging; OTC: Over-the-counter.

Individuals in the uPOD group will receive care typical of podiatric management of PHP at the Des Moines University Clinic that is in accordance with the recently updated practice guideline [20]. According to the guideline [20], the first 6 weeks of treatment includes foot taping/padding, home stretching exercises, over-the-counter arch support/heel cup, shoe recommendations, oral anti-inflammatories, and corticosteroid injections. If the participant is non-responsive to the first 6 weeks of treatment, the guideline indicates the next 6 months of treatment should include corticosteroid injections, custom orthotics, a night splint, immobilization, and referral to a physical therapist [20]. If the participant is not responsive after 6 months of treatment, extracorporeal shock wave therapy or a fasciotomy surgery is recommended per the guideline [20]. Extracorporeal shock wave therapy is not available at Des Moines University and partial or full fasciotomy is performed in less than 1% of patients. In addition to the treatment indicated above, the podiatrist may order radiographs or ultrasound imaging within their scope of practice. The specific intervention will be selected at the discretion of the treating podiatrist and Figure 2 indicates the prevalence of intervention and testing procedures from a random sample of care episodes between February 2008 and August 2011 at the Des Moines University Podiatry Clinic.

### Statistical analysis

Baseline group variables will be summarized using the mean and standard deviation for continuous measures and percentages for categorical measures. Independent *t*-tests (*P* <0.05, two-tailed) or the appropriate nonparametric test will be used to compare between-group differences in baseline characteristics (Table 1). Parametric test assumptions will be analyzed for all continuous variables by visual inspection of histograms, use of skewness score within double the standard error of skewness criteria, and Levene’s test for homogeneity of the variance. A repeated measures analysis of variance will be used to compare group differences in the FAAM, NPRS, EQ-5D™, and number of office visits associated with treatment at each time point. Results will be reported as the group mean, mean difference between groups, 95% confidence intervals, f-value, *P* value, power, and effect size. The χ^2^ test will be used to compare GROC scores between groups at each time point and results will be reported as the χ^2^ value, *P* value, and the frequency per category. The types of treatment based on Current Procedural Terminology and Healthcare Common Procedure Coding System codes provided per group will be reported as percentages. Analysis of covariance will be used if any group differences are observed in participant characteristics. Intention-to-treat analysis will be performed by comparing the complete case analysis to multiple imputation analysis. Multiple imputed data sets will be generated using the Multivariate Imputation by Chained Equations algorithm with SPSS 19.0 for Windows (SPSS Inc., Chicago, IL, USA). Multinomial logistic regression will be used to obtain pooled regression estimates from the data sets that will be used for analysis. Post-randomization exclusion of included participants that did not meet eligibility criteria or that did not receive intervention will be considered for exclusion of the analysis by an independent, blinded adjudication committee that will evaluate all randomized participants [57]. Any crossovers will be analyzed in the original group to which they were assigned. A separate analysis excluding crossovers will be performed.

Participant expectations and preferences for ePT or uPOD will be categorized into three categories; matched, unmatched, and neutral. Participants will be labelled ‘matched’ if they are allocated to a group for which they have expressed a higher expectation of benefit. A higher expectation will be denoted by comparison of visual analog ratings between the groups (ePT and uPOD; Additional file 1). Unmatched participants will be those who are allocated to the treatment group for which they have a lower expectation of benefit. Neutral participants will be those who indicate the same level of expectation for both treatments. Similar categorizations will be made for participant preference based upon their response to the treatment preference question (Additional file 1). General expectations of improvement will be dichotomized into met or unmet based upon rankings at the 6-week, 6-month, and 1-year follow-up relative to baseline rankings. Participants that demonstrate 6-week, 6-month, and 1-year ranks equal to or higher than baseline will be considered to have met their global expectations of improvement. Participants that demonstrate rankings below baseline expectations will be considered to have unmet global expectations of improvement. A χ^2^ test of independence will be used to compare the proportions of individuals with matched/unmatched/neutral expectations or preferences to global expectations (met or unmet). In addition, differences in expectation and preference categories will be analyzed relative to treatment success using a χ^2^ test of independence. Treatment success will be determined by the GROC at each period with success defined as a GROC of +5, “a great deal better”, or greater.

### Economic evaluation

An economic evaluation will be conducted alongside this randomized clinical trial in accordance with established methods [58,59]. Costs and consequences of ePT and uPOD from the viewpoint of a healthcare payer and society will be estimated using billing records and a participant-reported questionnaire at all follow-up time points. Incremental cost effectiveness will be reported using quality-adjusted life-years derived from the EQ-5D™ and index scores from the United States [37]. A separate protocol paper will describe the methods of the economic evaluation in greater detail and in accordance with recommendations from publication guidelines for economic evaluations [60].

## Discussion

This paper describes the methods of a randomized clinical trial comparing ePT and uPOD for PHP. The proposed work builds on emerging evidence indicating the benefit of early physical therapy intervention [27-29], which has not been established in a population of individuals with PHP. In addition, previous investigations have analyzed the outcome associated with individual or 1–2 combined interventions for PHP. This is the first pragmatic study, as far as we are aware, that investigates the role and cost-effectiveness of impairment-based physical therapy intervention early in the multi-disciplinary management of PHP. The implications of this work address national concerns about rising healthcare costs in the American healthcare system, will help inform clinical guidelines, and benefit multiple healthcare providers involved in PHP management. This trial is registered at clinicaltrials.gov, identifier NCT01865734 to increase transparency of trial. The results of this investigation will be submitted for publication in peer-reviewed journals and presented at local and national meetings regardless of the outcome. The Orthopaedic Section, APTA, Inc. will be credited for monetary support provided for this work in all publications and presentations.

### Limitations

This study will be the first to compare uPOD and ePT and this investigation will be conducted at one location, the Des Moines University Clinic. As a result, the findings may have limited generalizability to other settings. In addition, treatments provided by both groups will be done at the discretion of the provider. There will be six Podiatrists and four Physical Therapists providing treatment, which may result in variation of treatment within each group. Efforts will be made to reduce variation by providing a guide based upon current best evidence for the physical therapy intervention (Tables 2 and 3), but the intent of this trial is to capture the value and differences of pragmatic clinical approaches used to manage PHP. This may result in difficulty describing specific details about treatment beyond reporting of Current Procedural Terminology codes used when treatment is billed, but general aspects of treatment will be described in as much detail as possible.

The inclusion and exclusion criteria used in this investigation will help to reduce the likelihood of confounding factors but will also limit the generalizability of results. The results of this investigation may not apply to individuals with a BMI greater than 30 kg/m^2^, PHP symptoms for longer than 1 year in duration, older than 60 years of age, or comorbid conditions that were excluded in this trial (e.g., osteoporosis, rheumatic inflammatory diseases, severe peripheral artery disease).

All participants in this study will be seen by a podiatrist first and therefore the results of this investigation will apply to clinical scenarios when an individual presents first to podiatry with PHP. Preliminary unpublished investigation at the Des Moines University Clinic indicates that a significant majority of patients present first to podiatry than to any other specialty for PHP, but no other investigations were found to describe the prevalence of patient presentation to the primary providers for PHP. Therefore, the design of this trial is based upon local patterns of patient entry to the healthcare system for PHP. The results of this investigation may be limited in clinical scenarios where the patient may present to another provider first or does not receive an evaluation and treatment from a podiatrist initially.

## Abbreviations

ACFAS: American College of Foot and Ankle Surgeons; ADL: Activities of daily living; APTA: American Physical Therapy Association; BMI: Body mass index; ePT: Early physical therapy; EQ-5D™: European quality of life-five dimensions; FAAM: Foot and ankle ability measure; IRB: Institutional Review Board; MCID: Minimum clinically important difference; NPRS: Numeric pain rating scale; PHP: Plantar heel pain; uPOD: Usual podiatric care.

## Competing interests

The authors declare that they have no competing interests.

## Authors’ contributions

SMM is the principle investigator and will be responsible for the study design, IRB applications, participant recruitment, ePT treatment delivery, training of independent examiner who will collect data and compensate participants, and training of office and professional staff at the study location including analysis of baseline tests/measure reliability. He will also be responsible for data analysis and interpretation, presentation of results, and project write-up. TWF, BCH, and TGM will be responsible for the study design, data analysis and interpretation, presentation of results, and project write-up. DP and PAD will provide consultation of the design and analysis of the economic evaluation aspect of this study including manuscript writing. JDB will provide consultation on the podiatric-specific aspects of the study design including manuscript writing, and assist with implementation of study procedures in the Podiatry department including participating as a provider of uPOD treatment. All authors read and approved the final manuscript.

## Supplementary Material

Additional file 1 Participant expectation and preference questionnaire. Click here for file
