# Supplementary material for: Comparison of usual podiatric care and early physical therapy intervention for plantar heel pain: study protocol for a parallel-group randomized clinical trial
Source: Trials. 2013 Dec 3;14:414. doi: 10.1186/1745-6215-14-414 (PMC3866618; doi:10.1186/1745-6215-14-414)
Supplement: Additional file 1 — Participant expectation and preference questionnaire. [file 1745-6215-14-414-S1.pdf]

Additional File 1

Participant expectation and preference questionnaire

1. How do you expect your heel/foot pain to be in 6 weeks and in 6 months from now?

Please select only one for 6 weeks and one for 6 months	A very great deal worse	A great deal worse	Quite a bit worse	Moderately worse	Somewhat worse	A little bit worse	A tiny bit worse (almost the same)	About the same	A tiny bit better (almost the same)	A little bit better	Somewhat better	Moderately better	Quite a bit better	A great deal better	A very great deal better
**In 6 weeks**	☐	☐	☐	☐	☐	☐	☐	☐	☐	☐	☐	☐	☐	☐	☐
**In 6 months** (~26 weeks)	☐	☐	☐	☐	☐	☐	☐	☐	☐	☐	☐	☐	☐	☐	☐

2. The remaining questions pertain to expectations and preferences for 2 different approaches to treating plantar heel pain (aka, plantar fasciitis). When responding, consider Podiatry Care as treatment provided by a podiatrist (3-4 visits on average). One Podiatry visit, then physical therapy is one visit with a podiatrist followed by multiple physical therapy visits (6-8 on average). Consider physical therapy as treatment provided by a physical therapist (exercises done WITHOUT visits to a physical therapist is NOT considered physical therapy).

a. How helpful do you believe the following will be for this episode of your heel/foot pain? Please make one mark on each of the following scales.

Podiatry care

0 — 1 — 2 — 3 — 4 — 5 — 6 — 7 — 8 — 9 — 10

Not helpful at all | Extremely helpful

One Podiatry visit, then physical therapy

0 — 1 — 2 — 3 — 4 — 5 — 6 — 7 — 8 — 9 — 10

Not helpful at all | Extremely helpful

b. Which treatment would you chose to help your heel/bottom of foot pain (please select only one)?

☐ Podiatry Care ☐ Podiatry and early physical therapy intervention ☐ I do not have a preference
